# Guided discovery of chemical reaction pathways with imposed activation†

**DOI:** 10.1039/d2sc05135d

**Published:** 2022-11-10

**Authors:** Cyrille Lavigne, Gabe Gomes, Robert Pollice, Alán Aspuru-Guzik

**Affiliations:** Department of Computer Science, University of Toronto 214 College St. Toronto Ontario M5T 3A1 Canada gabegomes@cmu.edu r.pollice@rug.nl aspuru@utoronto.ca; Chemical Physics Theory Group, Department of Chemistry, University of Toronto 80 St George St Toronto Ontario M5S 3H6 Canada; Department of Chemical Engineering & Applied Chemistry, University of Toronto 200 College St. Ontario M5S 3E5 Canada; Department of Materials Science & Engineering, University of Toronto 184 College St. Ontario M5S 3E4 Canada; Vector Institute for Artificial Intelligence 661 University Ave Suite 710 Toronto Ontario M5G 1M1 Canada; Lebovic Fellow, Canadian Institute for Advanced Research (CIFAR) 661 University Ave Toronto Ontario M5G Canada

## Abstract

Computational power and quantum chemical methods have improved immensely since computers were first applied to the study of reactivity, but the *de novo* prediction of chemical reactions has remained challenging. We show that complex reaction pathways can be efficiently predicted in a guided manner using chemical activation imposed by geometrical constraints of specific reactive modes, which we term imposed activation (IACTA). Our approach is demonstrated on realistic and challenging chemistry, such as a triple cyclization cascade involved in the total synthesis of a natural product, a water-mediated Michael addition, and several oxidative addition reactions of complex drug-like molecules. Notably and in contrast with traditional hand-guided computational chemistry calculations, our method requires minimal human involvement and no prior knowledge of the products or the associated mechanisms. We believe that IACTA will be a transformational tool to screen for chemical reactivity and to study both by-product formation and decomposition pathways in a guided way.

## Abstract 2

Activation of a single user-selected coordinate such as stretching of a bond combined with a constrained conformer search *via* metadynamics allows chemical reaction pathways and the associated transition structures to be explored comprehensively.

## Graphical abstract

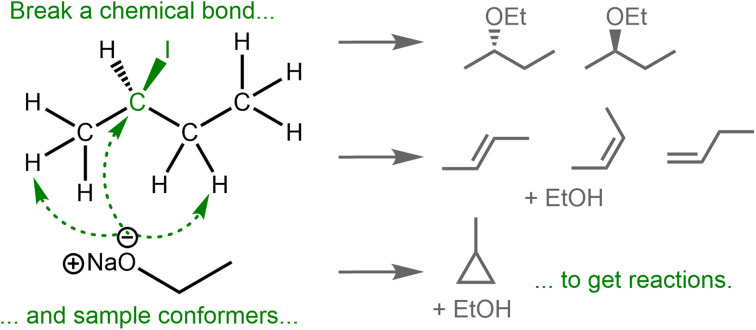

## Introduction

While a significant portion of the chemical reactions of scientific interest have half-lives on the order of seconds to days, the corresponding dynamic processes require time resolutions on the order of femtoseconds or even less for direct simulation. This difference of at least fifteen orders of magnitude in timescales makes brute force, large-scale computer simulations infeasible^1^ and, consequently, the prediction of chemical reactivity remains one of the most important challenges in computational chemistry. To tackle this challenge, many alternative approaches have been developed.^2^ In a recent review, these approaches have been classified with respect to the general strategy adopted for exploring potential energy surfaces.^3^ The three classes identified are (1) methods exploiting curvature information of the potential energy surface, (2) methods applying chemical heuristics, and (3) methods that rely on human intuition to guide the exploration.^3^ When methods combine multiple of these strategies they are classified based on the dominating one. Notably, we will limit this introduction to methods tackling forward explorations with an open end, *i.e.*, we know the identity of the substrates and wish to find out how they react.^4^ Thus, we exclude methods assuming known product states.

Among the approaches tackling open-ended forward explorations, heuristic approaches relying on human-derived rules for reactivity and stability^5–13^ are prominent and they largely belong to class 2 (*vide supra*). Predominantly mechanism-agnostic machine learning methods for product prediction based on experimental data^14–21^ are currently becoming increasingly popular and they formally belong to the same class as they simply utilize learned rather than human-derived rules. These approaches typically start with the prediction of likely intermediates and products allowing them to use methodology developed for start-to-end exploration^4^ thereafter for finding the corresponding reaction pathways.

In contrast, the prediction of potential reaction pathways starting from known minima leading to unknown products can also be tackled directly with *ab initio* simulations. In addition to the classification introduced above, the corresponding methods can be further grouped into several families. One of the simplest approaches is coordinate driving^22^ and it is one of the standard approaches used in contemporary user-guided potential energy surface exploration. The user decides on which, usually internal, coordinates are to be systematically modified and the corresponding energy profile is manually inspected. Accordingly, this method largely relies on human intuition and belongs to class 3. Based on the idea of coordinate driving, brute force approaches such as Grid Search,^22^ ValleyScan^23^ and the so-called single coordinate driving technique^24,25^ have been developed, which systematically explore all dimensions of the potential energy surface *via* exhaustive coordinate driving. Consequently, their computational expense increases steeply with the dimensionality of the system under study. These methods do not rely on human intuition anymore and solely utilize information from the potential energy surface which puts them into class 1.

To make exhaustive coordinate driving approaches more tractable, a combined molecular dynamics and coordinate driving (MD/CD) method^26^ has been developed which relies on molecular dynamics for systematic conformer sampling. Additionally, while still utilizing exhaustive coordinate driving for exploring reactivity, it allows users to define active atoms. Consequently, exhaustive coordinate driving is only applied to the active atoms thus reducing the dimensionality and scaling of the problem. Another variation of exhaustive coordinate driving has been realized in ZStruct2,^27^ which relies on the single-ended growing string method to construct reaction paths and find both transition states and the associated minima. It also allows for users to define active atoms to narrow down the space to be explored.

As an alternative to the previously discussed brute force approaches, class 1 methods like the gradient extremal walking algorithm (GEWA)^28^ and anharmonic downward distortion following (ADDF)^29^ directly exploit curvature information of the potential energy surface to identify paths leading to transition states and the corresponding connected minima. Nevertheless, exhaustive application of these approaches is still limited in scope and quickly becomes prohibitively expensive when the system size is increased. A different type of class 1 approach is the artificial force-induced reaction (AFIR) method.^30^ It applies an artificial force that pushes reactants together allowing reaction barriers to be overcome *via* ordinary energy minimization. By exploring many random initial reactant orientations, various alternative reaction pathways can be discovered. Notably, intramolecular reactions can also be explored by defining molecular fragments and user input can be utilized for the selection of suitable reactant pairs and active atoms within them.^31^

Arguably the most explored family of class 1 approaches relies on reactive molecular dynamics. Early examples include enhanced sampling techniques like metadynamics *via* local elevation^32,33^ and bias-potential-driven dynamics^34,35^ to overcome barriers between various conformers and between reactants and products. Additionally, reactions in molecular dynamics simulations can also be promoted by strong external electric fields,^36^ and by a virtual piston that periodically applies very high pressures to the simulated system to increase the frequency of collisions and thus the probability of barrier crossings, as realized in the so-called nanoreactor.^37^ The term nanoreactor has later also been used to describe metadynamics simulations of chemical reactions in confined spaces with time-independent wall potentials.^38^ Moreover, reactions can also be promoted by performing molecular dynamics with strongly vibrationally excited molecules as realized in the Transition State Search Using Chemical Dynamics Simulations (TSSCDS) approach.^39,40^ Notably, TSSCDS allows for the user-guided selection of vibrational modes to be excited to direct the reactivity to be explored.

Despite the plurality of existing approaches, current techniques for systematic prediction of chemical reactivity have significant drawbacks that curtail their general use, both in human-guided workflows and in fully automated settings.^3^ It is striking that many approaches for reaction pathway exploration show limited benefits from domain expert input. In fully automated settings, systematic and exhaustive reaction pathway searches are computationally prohibitive,^41^ and difficult to scale to larger molecules.^24,26,42^ Heuristics approaches can map extensive reaction networks^43^ but many of them in particular face issues when confronted with non-conventional bonding and organometallic species,^3^ which require alternative quantum chemistry-based strategies.^8^ Hence, there is room for a method that allows for both guided and systematic exploration of potential energy surfaces based on quantum chemistry simulations.

Herein, we introduce imposed activation (IACTA), a conceptually simple and general computational approach that semi-automatically and robustly explores chemical reaction pathways and their products. Additionally, it provides reasonable guess structures of the corresponding transition states. To do that, it requires as user input the identity of the substrates and selection of one reactive coordinate, which can be straightforward to choose for some systems but non-trivial for others, depending on the reactivity under study. Nevertheless, as the input does not require specific alignment of molecules or preliminary geometry optimization, it is operationally intuitive for human users and, in principle, also amenable to automated workflows. Specifically, we show that these pathways can be obtained by conformational exploration^38^ with a chemically activating constraint. This method provides meaningful suggestions for diverse reaction pathways together with an initial reaction feasibility assessment based on reaction energies and rough estimates of the associated barriers. Reactions of sizable molecules are found rapidly with only modest computational resources when employing state-of-the-art semi-empirical methods such as the GFN-xTB family.^44,45^ However, IACTA does not require the use of specific electronic structure methods and, thus, can be combined with any approach that computes energies based on 3-dimensional structures. Notably, while the separate ingredients of the workflow are well-established in computational chemistry, IACTA puts them together in a unique way that makes it both intuitive to use and computationally efficient by relying on user guidance. Despite introducing significant bias due to the requirement for user input, our results demonstrate that we are still able to discover many surprising reaction pathways. We present the capabilities of this approach on three main case studies with distinct challenges: (1) an epoxide-initiated stereoselective polyene tricyclization cascade with various side products, (2) a solvent-mediated Michael addition with a proton shuttle involving multiple water molecules, and (3) the late-stage oxidative addition of ten drug-like molecules with electronically unsaturated palladium phosphine complexes.

## Results and discussion

**Fig. 1:**
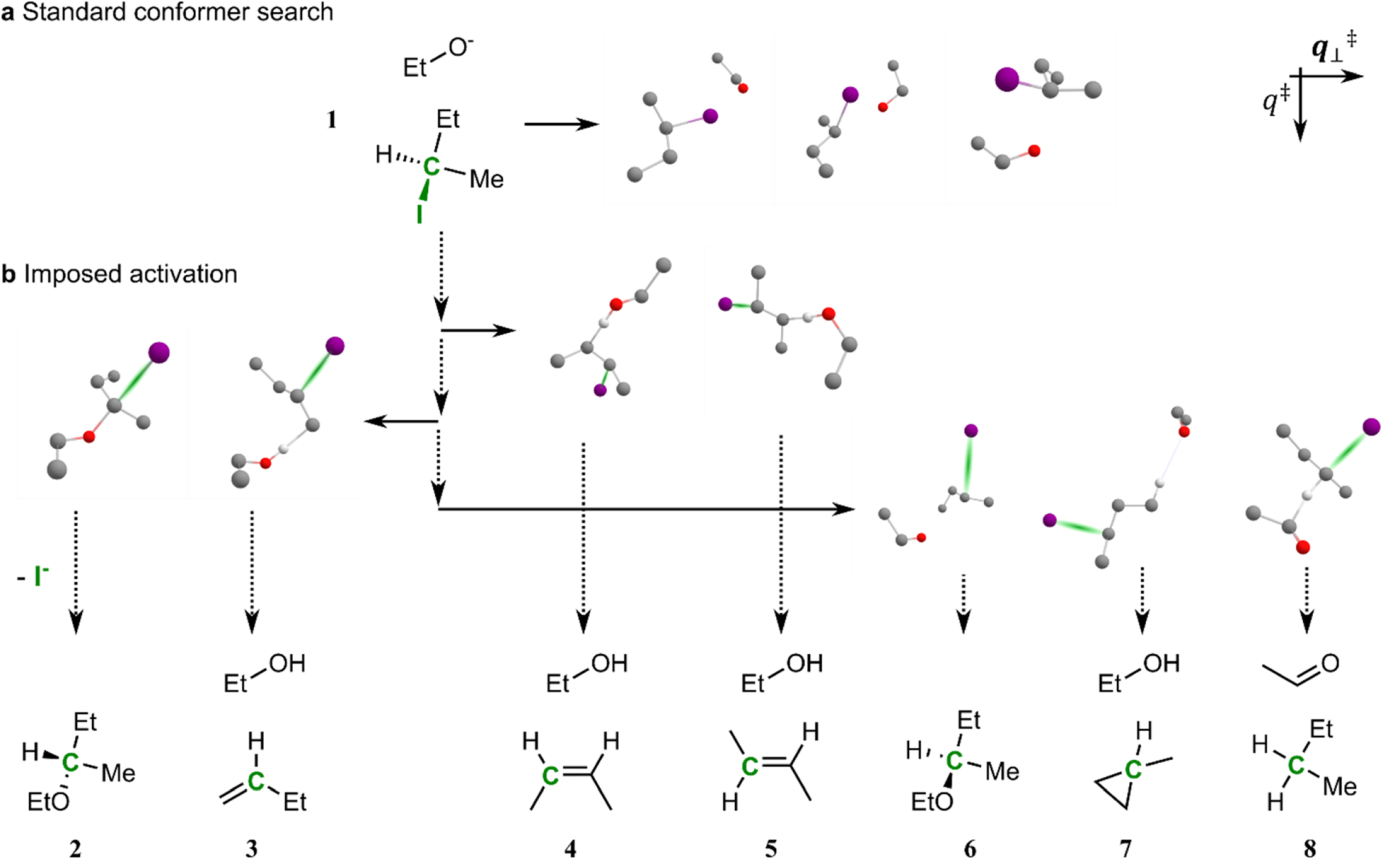
Diagram of reaction search by conformer exploration with imposed activation. (a) Conformer search methods generate stable three-dimensional molecular structures, such as those shown for complex 1, composed of a molecule of (*R*)-2-iodobutane and ethoxide anion. (b) Our reaction prediction methodology consists of constraining a specific activation coordinate *q*^‡^ to out-of-equilibrium values (vertical dotted arrows) and performing searches for activated conformers with the other coordinates unconstrained (solid horizontal arrows). This is demonstrated here on 1, using the carbon–iodine bond as *q*^‡^ (green). Further increasing the carbon–iodine distance of the activated conformers yields reaction pathways to multiple products (compounds 2–8). Non-reacting hydrogen atoms are omitted for clarity.

To initiate IACTA, a chemical coordinate (such as the length of a bond) is chosen as the activating coordinate *q*^‡^ (*cf.*Fig. 1). Subsequently, conformer generation^38^ is performed while constraining *q*^‡^ to a value between the initial reactant structure and possible product structures to explore the orthogonal coordinates *q*^‡^_⊥_. This corresponds to a search for local energy minima in the constrained space amidst the range of barriers separating products from reactants. These local energy minima, found by conformational exploration, are possible reaction pathways. A subsequent relaxed scan of *q*^‡^ pushes the molecule along discovered pathways to new products. We emphasize again that neither approximate transition structures, nor the identity of reaction products, nor any training data are required as input in the search, only the identity of the reactants and the choice of an activating coordinate. Overall, the workflow consists of four steps:

1. The user selects an activating coordinate *q*^‡^ (*i.e.*, an interatomic distance, a bending angle, or a torsional angle) and the initial structure of the reactants is optimized. With the reactive coordinate fixed at its equilibrium value *q*^‡^_0_, an initial conformer search is performed to obtain a diverse set of structures. A relaxed scan of the reactive coordinate follows, starting with one of the initially generated conformers, to an intermediate value between that of reactants and products *q*^‡^_i_.

2. A second, shorter and less exhaustive conformer search is undertaken with the reactive coordinate constrained at *q*^‡^_i_ to explore the local energy landscape.

3. All the structures thereby generated are scanned from *q*^‡^ = *q*^‡^_i_ to *q*^‡^_N_, with *q*^‡^_N_ being the maximum scanned value, to obtain new reaction products. A backward scan from *q*^‡^_i_ to *q*^‡^_0_ yields a complete trajectory back to a reactant conformation.

4. Finally, structures corresponding to energy minima of the trajectory (potential reactants and products conformations) are optimized without constraints to ensure stability.

Steps 2 to 4 above are then repeated for different values of *q*^‡^_i_ to obtain an ensemble of reactive trajectories sampled from multiple transition structures.

As a concrete example, let us consider the various elimination and substitution reactions of (*R*)-2-iodobutane and ethoxide anion. Generating ground-state conformers for this system yields many loosely bound complexes (Fig. 1a). Reaction prediction starting from these structures following specific normal modes is challenging, as they are quite far from any connected transition states, such as those shown in Fig. 1b.

One standard approach used extensively by methods like coordinate driving and related approaches to traverse the mountain ranges of potential reactive modes is to pull together pairs of atoms *via* relaxed scans to form new bonds.^26,42,46^ Thus, they circumvent the issue of initial structures being too far from transition states by gradually guiding them into reactive conformations. For example, pulling together the ethoxide anion oxygen and the α-carbon of 2-iodobutane leads to the S_N_2 reaction. However, for predicting potential reaction outcomes without prior knowledge about which pairs of atoms are likely to react, a fully systematic search of all possible bond-forming pairs is necessary which scales quadratically with the number of atoms^26^ and generally can only be performed for relatively small systems.^47^ Additionally, if prior knowledge is available, this approach requires information about multiple sites that could potentially react with each other.

The key idea of our approach is to activate reactants using a single coordinate *q*^‡^ that can initiate many reactions of interest, thereby circumventing the scaling issues of searching for possible bond formation pairs systematically or heuristically. In the case of the (*R*)-2-iodobutane molecule depicted in Fig. 1, bond dissociation energies dictate that the carbon–iodine bond is readily broken,^48^ making it an excellent choice for *q*^‡^. Stretching the carbon–iodine bond, *i.e.*, increasing *q*^‡^, creates activated and very electrophilic structures. A subsequent constrained conformer search in the orthogonal space *q*^‡^_⊥_ yields geometries close to the S_N_2 transition state, as the ethoxide oxygen stabilizes the structure when it lies close to the halogenated carbon. Relaxed scans along *q*^‡^ starting from one of these newly generated transition structures leads to the S_N_2 products (2 in Fig. 1).

Importantly, besides S_N_2, in a single run of our workflow, other activated conformers leading to different reaction products are also uncovered. Stretching the carbon–iodine bond of all the activated structures found also yields E_2_ and E_1_ elimination products (with both Hofmann^49^ product 3 and Zaitsev^50^ products 4 and 5), S_N_1 substitution reactions (with products 2 and 6), γ-elimination to methylcyclopropane 7 as well as a curious alkoxide hydride transfer^51^ forming 8. The latter two reactions, which have significantly higher estimated reaction barriers, are unlikely to occur in an experiment but, first, do represent known chemistry and thus are still reasonable, and second, demonstrate the complex pathways that can be obtained automatically from imposed activation with a single activating coordinate.

The reaction pathways discovered in coordinate scans are highly dependent on the corresponding starting geometries, especially when the scanned coordinate is not bond-forming. Indeed, for an iodine–carbon bond scan to generate a pathway towards compound 7*via* γ-elimination requires the ethoxide oxygen in very close proximity to the eliminated hydrogen, whereas it needs to be close to the α-carbon to proceed to the S_N_2 product 2. The diversity of products and reaction paths discovered by IACTA is made possible by bringing together two key concepts in a unique way. First, the activation of a single coordinate such as bond stretching generates a highly reactive species that can, in principle, undergo many transformations downhill in energy depending on its conformation. Second, the power of constrained conformer generation *via* metadynamics explores diverse reactive conformations of this species^38,52^ which are directly connected to a wide range of reaction pathways allowing subsequent relaxed scans to find new reaction products. Finally, these concepts are made practical and applicable to reasonable large molecular systems by relying on the computational efficiency and robustness of the GFN-xTB family of methods as computational workhorse.^45^ However, we would like to emphasize that IACTA does not require use of this specific family of methods but can be combined with any approach predicting energies from 3-dimensional structures.

To summarize the key features of IACTA and compare them with the main classes of alternative approaches, we again rely on the framework consisting of three distinct classes that was established in a recent review (*vide supra*).^3^ Specifically, we not only consider the main class of each method but also the extent to which they incorporate ideas of the other classes. Additionally, we assess how these approaches fulfill the main features demanded from systematic methods for potential energy surface exploration, as proposed in the same review.^3^ The corresponding results are summarized in Table 1. IACTA makes use of both curvature information and human intuition to explore reactivity and combines elements from both coordinate driving and reactive molecular dynamics approaches. Incorporation of heuristics, while currently not implemented, is possible, especially when aiming for a fully automated implementation testing alternative activating coordinates autonomously. Importantly, IACTA provides tractability at the cost of considerable thoroughness by relying on guidance based on user input. This provides IACTA with a prominent interactive component, unlike many alternative approaches. Overall, universal applicability of IACTA would require an automated method for selecting activating coordinates that could optionally supplement user choices enabling more thorough exploration. However, increasing thoroughness of IACTA will necessarily come at the cost of tractability.

**Table 1:** Comparison of algorithms for the exploration of chemical reaction pathways based on a framework proposed in the literature 3a

Feature	Coordinate driving	Exhaustive coordinate driving	AFIR	Reactive molecular dynamics	IACTA
Curvature information	✓	✓	✓	✓	✓
Heuristics	✗	∼	∼	∼	∼
Human intuition	✓	∼	∼	∼	✓
Full automation	✗	✓	✓	✓	∼
Thoroughness	✗	✓	✓	✓	∼
Tractability	✓	∼	∼	∼	✓
Guidance	✓	∼	∼	∼	✓
Universal applicability	✗	∼	∼	∼	∼

a ✓ and ✗ indicate the presence and absence of a feature, respectively. ∼ indicates that realization of a feature within the algorithm is, in principle, possible but only with compromises.

Apart from the reactions between (*R*)-2-iodobutane and ethoxide anion presented in Fig. 1, we used IACTA to explore reactivity in a diverse sample of similarly small molecules. The corresponding results are detailed in the ESI.† In the following sections, we apply IACTA to several more challenging examples inspired by recent literature studies to exemplify how IACTA can be applied to solve problems in chemistry.

### Cyclization cascade in the synthesis of a natural product

**Fig. 2:**
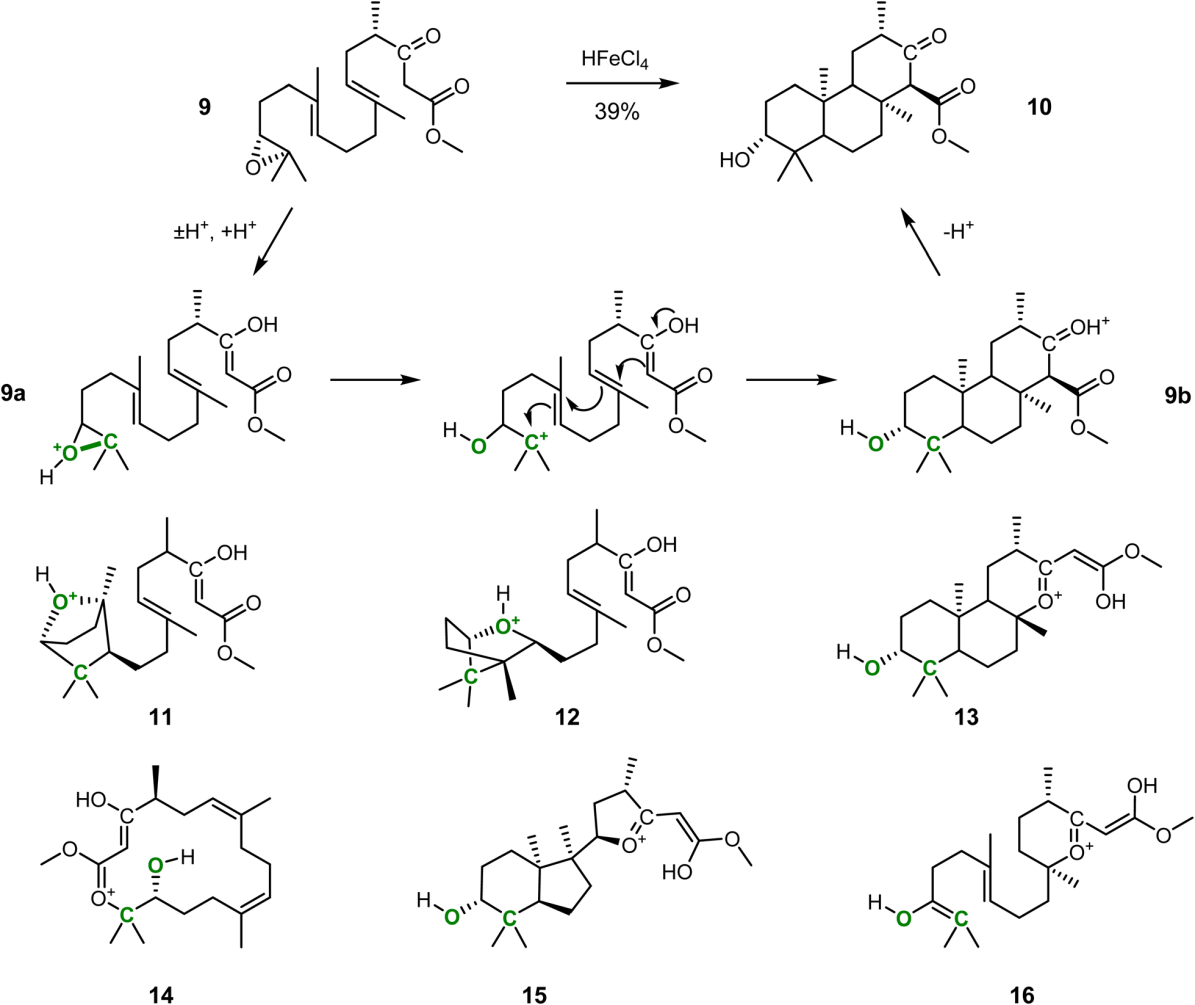
Epoxide-initiated β-keto ester-terminated triple cyclization reaction and the corresponding products and side-products predicted by IACTA. The polycyclization from 9 to 10 is a key step in a concise total synthesis of berkeleyone A described in the literature.^53^ We initiated an IACTA reaction search from protonated enol 9a using the epoxide C–O bond of the tertiary carbon indicated in green as the activating coordinate. Compounds 11–16 are a subset of the predicted side products.

A robust and automated reaction prediction approach can help to understand and rationalize complicated reactions with multiple outcomes. We demonstrate this based on the epoxide-initiated polycyclization of 9 to form 10 (*cf.*Fig. 2), an essential step in a concise total synthesis of the fungal meroterpenoid berkeleyone A.^53^

We selected this triple cyclization as it is a complex reaction, with the potential to form many by-products.^54–57^ Indeed, the yield of 39% reported in the literature^53^ was only achieved after extensive optimization of reaction conditions and hints towards significant by-product formation. Previous attempts at similar reactions yielded a range of mono- and polycyclic, fused and bridged products from ether formation by the epoxide and cyclization at the β-keto ester oxygen instead of the desired carbocyclization.^58^ We initiate IACTA from structure 9a, the reactive enol form of 9 protonated at the epoxide. Compound 9a is activated by opening the epoxide on the tertiary carbon side using the carbon–oxygen distance as the activating constraint (*cf.*Fig. 2, bond highlighted in green). More than 200 distinct reaction pathways are discovered, including many tautomerizations, high energy processes, and, most importantly, all experimentally reported modes of action.

**Fig. 3:**
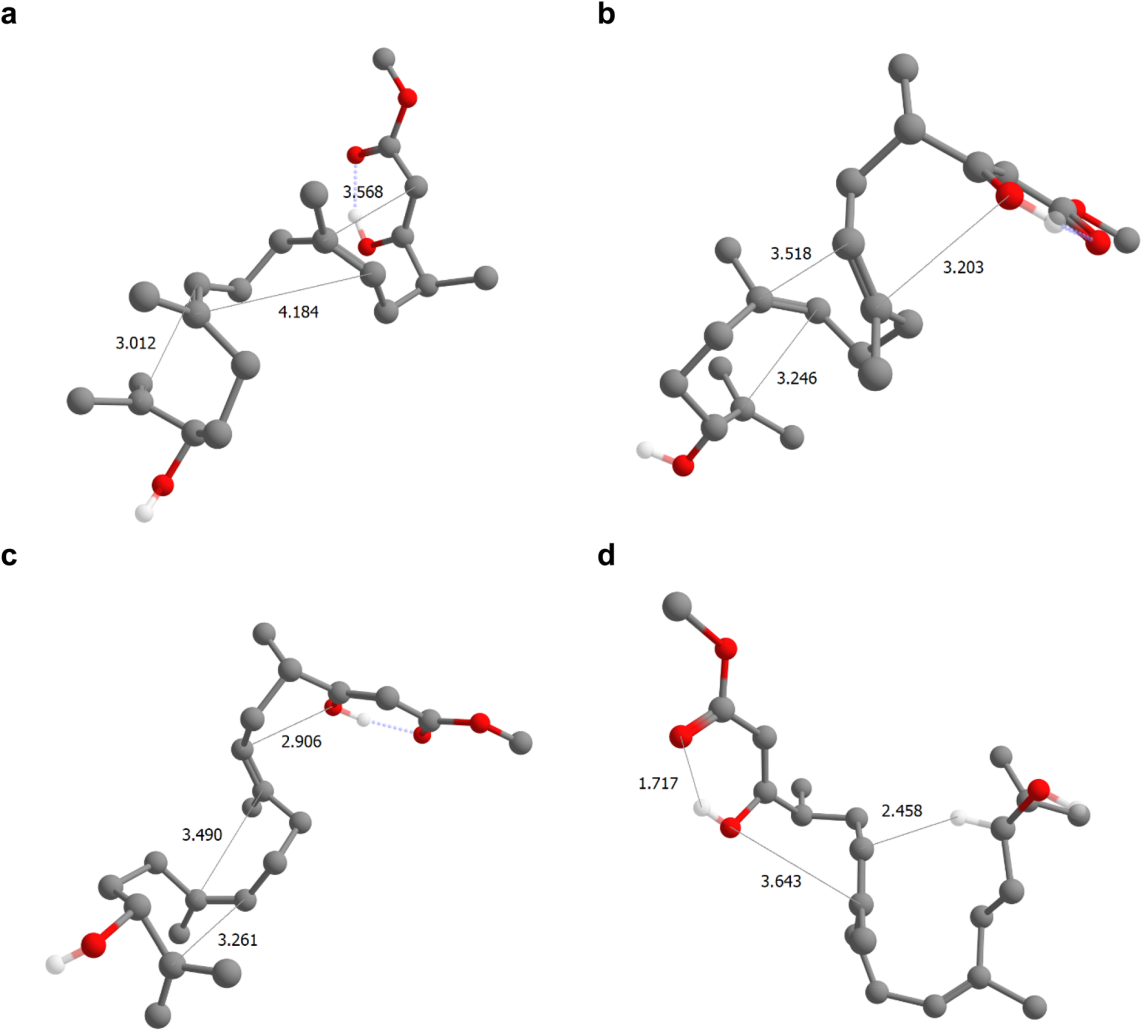
Selected transition structures found in the IACTA simulation of the epoxide-initiated reactions of berkeleyone A forming triple cyclization products. (a–d) Transition state guesses for the formation of 9b (a), 13 (b), 15 (c) and 16 (d). Bond-forming atoms are connected by thin lines annotated with their interatomic distances. Non-reacting hydrogen atoms are omitted for clarity.

A subset of the thermodynamically favorable products with estimated activation energies below 35 kcal mol^−1^ is shown in Fig. 2. Notably, 9b is readily discovered by the reaction search from an epoxide-initiated triple cyclization cascade terminating at the β-keto ester which forms the desired product 10 after deprotonation. The transition structure, *i.e.*, the transition state guess obtained from IACTA, for this reaction is depicted in Fig. 3a, with the three newly formed bonds shown with thin lines. We find this cyclization cascade to be barrier-free after this transition structure. Side products 11 and 12 result from 1,2-addition of the epoxide to the nearest alkene, forming bridged bicyclic ethers.^54^ Triple cyclization terminating at the β-keto ester oxygen forms 13, a major experimental by-product,^58^*via* a pathway that differs from the one forming 9b only by the nucleophilic atom initiating the cascade (*cf.*Fig. 3b). Direct nucleophilic epoxide opening by the same oxygen yields 14, which has a 16-membered ring. Product 15 is obtained like 13 except for the second stage of the cascade being formation of a five-membered rather than a six-membered ring (*cf.*Fig. 3c). Finally, the most surprising of the predicted products is 16, which is formed from the transfer of a proton from the epoxide (which forms an enol^55^) to the far alkene followed by an oxygen cyclization (Fig. 3c).

The straightforward initialization and short duration of our computational experiments employing IACTA are one of its crucial features. All the above reactions were obtained from a single run targeting compound 9a, which took only 6.3 hours on a single compute node. In this case, however, we note that success followed a failed search initiated from the less reactive keto form of 9a. Similarly, computations starting from another tautomer of 9, namely the enol of the ester, uncovered significantly fewer reactions and, to our surprise, failed to yield 9b. In practice, the sensitivity to the initial structures was solved by iteratively trying new tautomeric structures, an approach enabled by the fast turnaround times of the IACTA calculations, and the ease of tautomer generation using basic chemical rules,^59^ also making it amenable to automation.

### Water-mediated Michael addition

Automated reaction search is specifically needed when the reacting system consists of multiple loosely bound fragments with many possible arrangements. This is the case, for example, in solvent-mediated reactions, where specific solvation geometries play a major role in enabling various transition structures.^60^ Setting up traditional transition state calculations for such systems is very time-consuming and error-prone, as it requires carefully arranging fragments in various sometimes non-intuitive reactive geometries.^61^

**Fig. 4:**
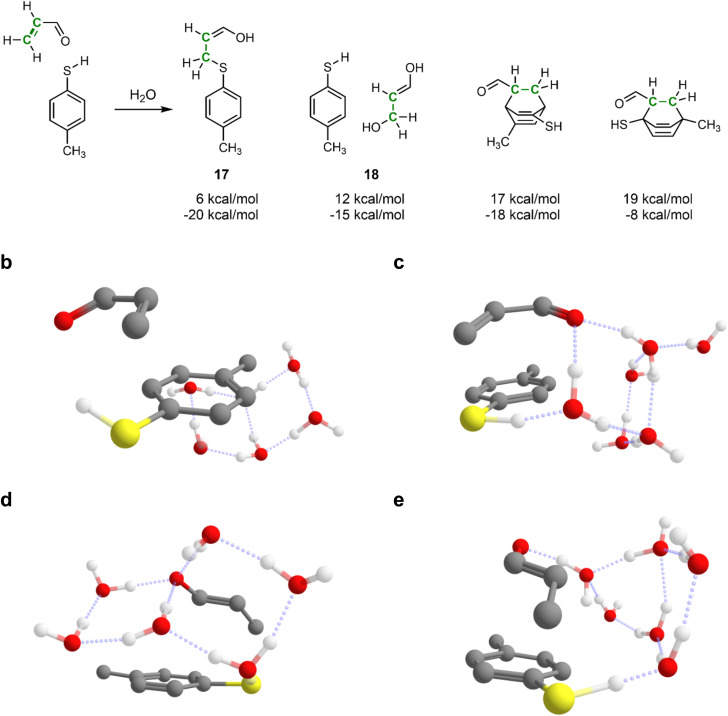
Water-catalyzed 1,4-addition reaction between acrolein and 4-methylthiophenol and some important side products predicted by IACTA. (a) Reactions were obtained by stretching the carbon–carbon double bond of an acrolein molecule (shown in green) in the presence of 4-methylthiophenol and six explicit water molecules. Predicted products are annotated with estimated activation energy (top) and reaction energy (bottom) at the GFN2-xTB level of theory. (b–e) Reaction pathways forming 17 include a direct proton transfer (b) and proton transfer shuttled by one (c), two (d), and four water molecules (e). Structures (b) and (c) are *s-cis* and structures (d) and (e) are *s-trans*, and all of them are compact conformations.

Therefore, next, we demonstrate IACTA on the water-mediated Michael addition of *p*-methylthiophenol to acrolein, shown in Fig. 4a, a case study inspired by a recent popular science article.^62^ Following this article, six explicit water molecules were added to the reacting system. Notably, as will be apparent from the following paragraphs, this choice of the explicit number of solvent molecules is non-trivial and strongly affects the outcome of the simulations. The addition reaction forming 17 was obtained as both thermodynamic and kinetic product upon stretching the carbon–carbon double bond of acrolein. By-products include hydration of acrolein to 3-hydroxypropanal (18), a biologically important process,^63^ and various Diels–Alder reactions between acrolein and 4-methylthiophenol that break aromaticity of the latter with higher estimated barriers.

This Michael addition involves proton transfer from the thiol to the carbonyl group of acrolein, which is facilitated in the simulations by a chain of hydrogen-bonded water molecules. Acrolein can have *s-cis* or *s-trans* configuration, and the reaction proceeds *via* the acrolein π-system either pointing towards the aromatic ring of the thiophenol (compact conformation) or away from it (expanded conformation). Among all the combinations of acrolein configurations and conformers, and the number of chained water molecules, IACTA identifies in a single run fourteen different pathways and distinct transition structures (Table 2), five of which are estimated to be faster than formation of 18. Thus, our method is excellent for screening potential pathways before embarking on extensive transition state optimization.

**Table 2:** The 176 reaction trajectories found yielding 17, classified based on transition structure characteristics^a^

Acrolein isomer	TS conformation	Number of H_2_O molecules in H^+^ transfer chain				
		0	1	2	3	4
*s-cis*	Compact	21.2|6	**8.7|20**	**11.2|8**	—	—
	Expanded	—	15.6|17	13.6|5	17.1|1	—
*s-trans*	Compact	—	27.4|1	**5.8|49**	**8.5|14**	20.6|1
	Expanded	—	23.3|4	**9.8|41**	17.1|11	16.6|1

a For each entry, the estimated activation energies at the GFN2-xTB level of theory in kcal mol^−1^ (bold, <12 kcal mol^−1^) and the number of occurrences are provided. Estimated Δ*E*^†^(kcal mol^−1^)|no. of occurrences.

Reaction without hydrogen-bonding water molecules facilitating the proton transfer only occurs for the *s-cis* isomer, as it requires the carbonyl and thiol to be in proximity (Fig. 4b). The longer distance required for the proton transfer in the *s-trans* isomer favors proton shuttling by two or three water molecules. In contrast, addition of the *s-cis* isomer is efficiently mediated by one water molecule (Fig. 4c). The lowest activation energy is obtained for the *s-trans* isomer facilitated by two water molecules in the compact conformation (Fig. 4d). The longest identified water chain acting as proton shuttle comprises four water molecules (Fig. 4e) with a total of five distinct proton transfers. In the expanded conformation, only *s-trans*-acrolein with a two-water molecule bridge is favorable. We note that while computational studies can overemphasize the importance of intramolecular proton shuttles due to the difficulty of treating solute–solvent proton transfer steps,^64^ this is not a problem specific to IACTA but rather a general characteristic of computations not representing bulk solvent in an explicit manner.

Importantly, IACTA provides useful insight into complicated systems such as this Michael addition requiring minimal human labor at the cost of comparably moderate computational resources. Indeed, sampling of these pathways and the corresponding transition structures *via* IACTA took only 20 hours of wall-clock time on a single compute node. The search is fully automated and is successful even when starting from a structure far from the identified reactive pathways. While the obtained results in terms of estimated reaction barriers are only preliminary as they only stem from semi-empirical methods and are not based on actual transition states, the obtained energies can be used for high-throughput virtual screening^65^ as a qualitative guide to estimate the feasibility of the predicted pathways. Additionally, the corresponding transition structures are reasonable starting points for subsequent transition state optimization, offering significant potential to reduce the associated human labor compared to traditional approaches.

### Oxidative addition complexes of drug-like compounds

In addition to the exploration of reaction pathways and the prediction of potential by-products, IACTA can also reduce the human effort for setting up transition state calculations and diminish the entrance barrier to computational tools in chemistry by providing reasonable guess structures for subsequent transition state optimizations. We demonstrate this based on calculations for the oxidative addition of a palladium catalyst to a set of drug-like molecules.^66^ As demonstrated in the literature, forming oxidative addition complexes in this way provides a powerful means for late-stage diversification in experimental screening studies and avoids the sometimes inconsistent performance of catalytic Buchwald–Hartwig couplings with highly functionalized substrates.^66^

**Fig. 5:**
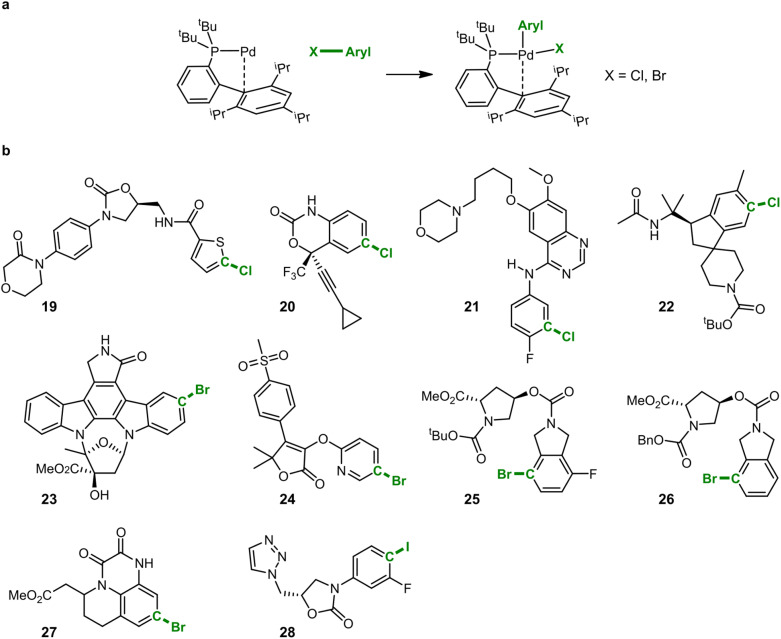
Formation of oxidative addition complexes of drug-like substrates. (a) The reaction studied here is the formation of Pd(ii) complexes from ^*t*^BuXPhosPd by oxidative addition. The aryl-halide bond is the activating coordinate which is depicted in green. (b) IACTA is performed for the ten drug-like, highly functionalized molecules 19–28.

Computational studies of organometallic catalysis typically tackle either complex ligands or complex substrates, but very rarely both, likely due to the required computational resources. Such studies are specifically challenging when revolving around designer catalysts such as ^*t*^BuXPhosPd, which we studied here (*cf.*Fig. 5a), and functionalized ligands, due to the many possible coordination modes with the metal center.^67^

**Fig. 6:**
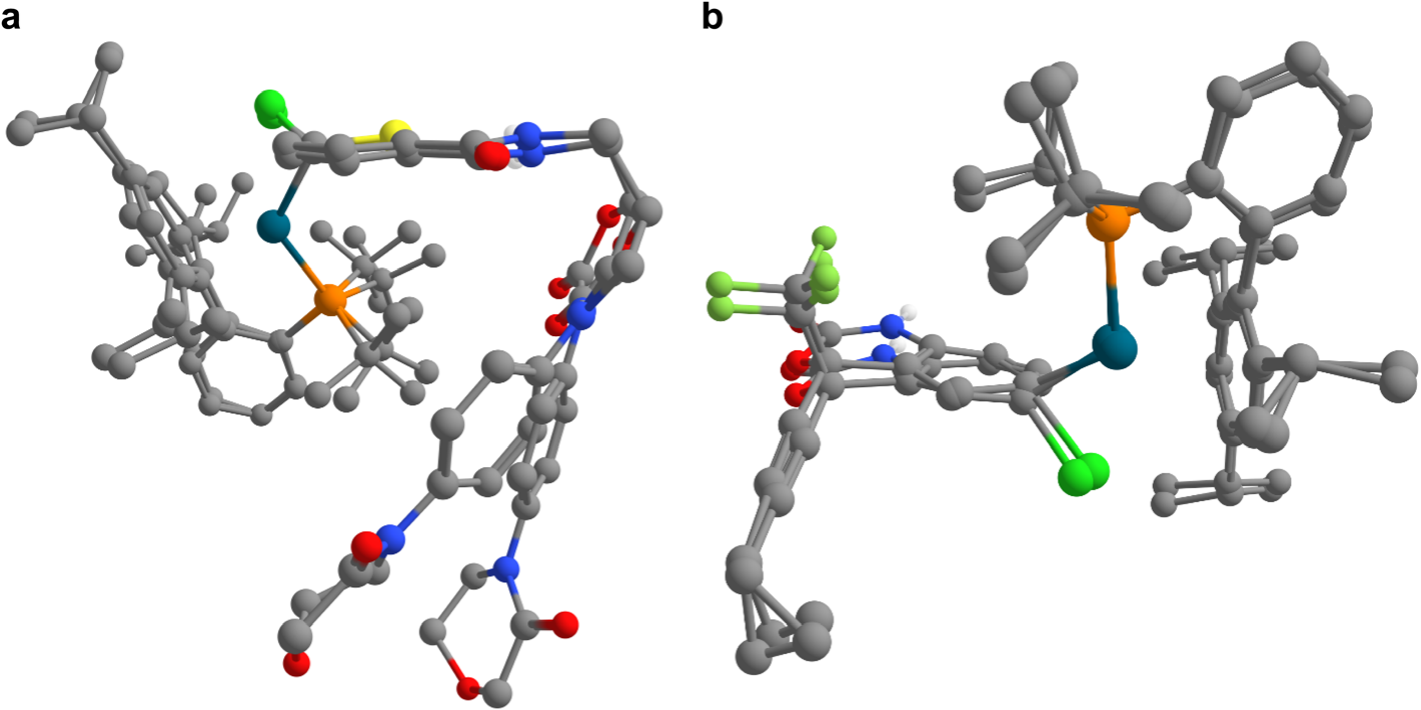
Superimposition of approximate transition structures obtained from IACTA at the GFN2-xTB level of theory with optimized transition state structures at the PBE-D3/def2-SVP level of theory for the formation of oxidative addition complexes of drug-like substrates. (a and b) Transition structures are shown for compounds (a) 19 and (b) 20. Notably, the GFN2-xTB transition structures are obtained directly from IACTA, without additional computational refinements.

In this case study, we present transition state calculations enabled by IACTA for ten reactions with both complex catalysts and complex substrates. Because the aims of this section differ from those of the previous two (*i.e.*, generating good transition state guess structures for a predetermined reaction as opposed to searching for new reactions), the calculations were set up in a more systematic way. Using automated structure generation, the activated catalyst was initially placed such that the Pd atom is approximately 5 angstroms away from the target halide. The halide-aryl bond was chosen as activating coordinate. The IACTA workflow was initiated with this structure and only the most accessible reaction pathway was selected. Finally, the lowest-energy reactant and product structures were re-optimized at the PBE-D3/def2-SVP level of theory. Transition states were obtained by relaxing the automatically obtained approximate transition structures at the PBE-D3/def2-SVP level of theory while constraining the aryl–halide bond, followed by a subsequent transition state optimization *via* the nudged elastic band method. Qualitative features of the transition states are well-reproduced by IACTA at the GFN2-xTB level of theory, as illustrated in Fig. 6.

The corresponding results are summarized in Table 3. The data show two general trends. First, as expected, activation energies tend to decrease when changing the halogen from chlorine to bromine to iodine.^68,69^ Secondly, electron-rich (hetero)aromatic rings seem to lead to more facile oxidative addition. This is in contrast to the expected trends for bisligated palladium(0) catalysts but in line with the results obtained for monoligated palladium(0), as the pre-reactive complexes of monoligated palladium(0) and aryl halide tend to be more tightly bound for electron-poor (hetero)aromatic rings.^69,70^ Furthermore, oxidative addition products with electron-poor (hetero)aromatic rings tend to be more thermodynamically favorable, as previously demonstrated.^70,71^ Moreover, all the estimated oxidative addition barriers are consistent with facile room temperature reactions, as observed in the original study.^66^ Looking at the contributions of London dispersion to the reaction energies, we find that larger Gibbs free energies of activation correlate with more repulsive dispersion contributions. This indicates that attractive interactions are lost during the oxidative addition, causing an overall higher barrier, and suggests that proper alignment of noncovalent interactions in the transition states is important in accelerating these reactions.^72,73^ While none of these findings in and of themselves are novel, the key point we wish to convey is the operational ease of obtaining these results with the help of IACTA.

**Table 3:** Summary of IACTA calculations and transition state optimizations for the oxidative additions shown in Fig. 5a^a^

Substrate	*N*_atoms_	Halogen	Core aryl group	Reaction energies (kcal mol^−1^)		Compute time (node hours)	
				Δ*G*^‡^	Δ*G*	IACTA	DFT optimizations
19	123	Cl	Thiophene	13.5	−12.2	10.6	4.5
20	106	Cl	Benzoxazinone	8.9	−16.7	4.8	5.1
21	131	Cl	Benzene	16.0	−6.7	10.3	3.7
22	141	Cl	Indane	4.4	−15.8	10.5	4.5
23	132	Br	Indole	1.1	−18.1	8.7	4.1
24	118	Br	Pyridine	12.2	−12.1	7.6	3.9
25	130	Br	Isoindoline	7.6	−9.6	9.6	4.1
26	131	Br	Isoindoline	8.0	−5.6	10.7	4.6
27	110	Br	Quinoxalinedione	4.7	−16.2	5.2	6.1
28	106	I	Benzene	6.0	−2.1	4.9	4.3

a The first four columns provide the substrate identity, the total number of atoms in the system, the reacting halide identity, and the reacting aromatic system on the substrate. Gibbs free energies of activation Δ*G*^‡^ and Gibbs free energies of reaction Δ*G* are obtained from PBE-D3/def2-SVP calculations. The last two columns compare the compute times required (in node hours) for IACTA and subsequent DFT optimizations of product, reactant, and transition state geometries.

Finally, we show in Table 3 that the computing demands of IACTA using GFN2-xTB are about twice as large as those of the subsequent PBE-D3/def2-SVP geometry optimizations, and thus are well within the realm of current high-throughput virtual screening capabilities^74^ allowing to perform reaction screening on a significantly larger scale than demonstrated here.

## Conclusion and outlook

Virtual reaction prediction has significant potential to accelerate the search for novel catalysts, automatically flag detrimental reaction pathways (*e.g.*, solvolysis or formation of side products), and help discover new reaction mechanisms. However, chemical reactions are extremely rare events not spontaneously captured by *in silico* dynamics. The complexity of all but the smallest molecules limits full, systematic searches of all possible bonding patterns and bonding transformations.

In this work, we showed that the combination of two key concepts, namely the activation of a single coordinate such as bond stretching chosen by the user and the power of constrained conformer generation *via* metadynamics, is the foundation of a powerful method to explore chemical reaction pathways and provide the corresponding transition state guess structures. When implemented with robust and efficient semi-empirical quantum chemistry methods such as the GFN-xTB family of methods, it only requires modest computational resources while still providing meaningful results. Nevertheless, using IACTA with alternative electronic structure methods is straightforward. Leveraging user input for the selection of activating coordinates such as a bond to be broken allows us to bypass the quadratic scaling of all possible bond-forming processes encountered by more systematic approaches and explore pathways to be most likely for a given set of substrates or to be of most interest in a given situation. In essence, IACTA is analogous to starting a curly arrow mechanism by breaking one bond, and then computationally completing the mechanism by systematically finding the most energetically feasible responses. However, this comes at the cost of losing some rigor for the systematic exploration of reaction pathways by introducing user bias.

Importantly, while IACTA can in principle be combined with any level of theory, the use of IACTA with semi-empirical methods is sufficiently low-cost to be deployed in an exploratory fashion for medium-to high-throughput virtual screening, predicting, and studying the mechanisms behind by-product formation or catalyst decomposition, and exploring the role of explicit solvation in chemical reactions. Although the semi-empirical calculations used in this work are appropriate for qualitative discovery, they are not accurate enough for quantitative analysis.^52^ Yet the high degree of inherent parallelism present in our approach makes it scalable on distributed and cloud compute systems, which would compensate for the computational demands required even when higher levels of theory are employed.^75^ Alternatively, to increase the computational accuracy without sacrificing computational efficiency, machine-learning based computational chemistry methods such as the ANI family of methods^76,77^ offer huge potential for even more reliable exploration of reactivity together with IACTA. We are currently investigating the use of machine learning methods (such as Gaussian process regression^78–80^) to robustly refine the transition state guesses obtained from IACTA. Investigations are also underway to improve systematicity by automating the selection of potential activating coordinates. This could enable the study of complex chemical reaction networks^3,81^ by applying IACTA recursively over obtained products.^43^ Finally, the inclusion of automated (de)protonation and tautomerization capabilities^82,83^ would increase the range of chemistry that can be studied systematically by IACTA out of the box and we are testing their implementation as well.

Overall, we believe that IACTA sets the stage for a new approach to computational investigation of reactivity. Accordingly, we envision IACTA to be an important stepping stone towards a true virtual lab where reactivity can be explored in a similar manner as it is done in an experimental lab, without knowledge of the outcome, providing valuable ideas for subsequent in-depth investigations and entire research campaigns.

## Methods

The results presented in this paper were obtained using the xTB package^45,84^ (version 6.3.0) and a custom interfacing and data analysis code. Additional results for the Buchwald–Hartwig coupling reactions were computed using ORCA (version 4.2.1),^85^ as described below. Timing data is reported for a single dual-socket compute node with two 20-core, 2.4 GHz Intel Skylake processors.

## Algorithmic details

For generating conformers, the metadynamics module integrated in xTB is used with parameters based on those of the highly reliable algorithm of Grimme *et al.*^38^ In effect, molecular dynamics are performed with regular snapshots, and these simulations are augmented with a biasing potential 
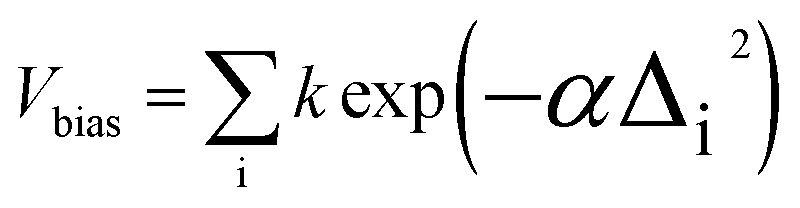
 where Δ_i_ is the root-mean-square displacement (RMSD) between the current structure and the i-th previous snapshot, and *k* and *α* are numerical parameters. The biasing potential strongly drives the discovery of new structures. The obtained structures are then optimized, with similar structures being removed based on RMSD, rotational constant and energy thresholds.^38^ Finally, structures exceeding a maximum energy threshold are discarded. Numerical values for all parameters are given in the ESI.†

In the examples presented, the overall number of sampled trajectories varies between a few hundreds (such as the iodobutane example) and 10 000 (to obtain the results of Table 2), depending on the number of atoms and the chosen sampling parameters. The duration of metadynamics simulations, and thus the number of trajectories, is chosen proportional to the size of the system. A more thorough sampling is done for the 1,4-addition to explore many reactive geometries.

For post-processing, the obtained trajectories are collected and parsed into reactions by transforming geometries of trajectory minima to canonical SMILES using Open Babel^86^ and identifying changes in the SMILES strings. For structures containing a metal complex, the metal atom is removed before conversion to eliminate spurious bond changes. The transition structure of a trajectory is taken to be the highest energy structure between reactants and products. The guess transition state for a given reaction is the lowest energy transition structure amongst all trajectories for this reaction, and the estimated activation energy is computed by subtracting the energy of the most stable identified reactant conformer from the energy of the approximate transition state.

### Refinement of trajectories for oxidative addition complexes

Additional refinement of reaction trajectories was performed for the results shown in Table 3. The specific protocol described here broadly follows that of the computational study by Barder *et al.* involving a similar catalyst.^87^ We note that our study involves more substrates and that those substrates are more complex. DFT calculations were performed at the PBE-D3/def2-SVP level of theory.

Approximate transition structures obtained from the reaction search were used to find transition states with Berny optimization at the GFN2-xTB level of theory. Here, performance is excellent, with every guess transition structure converging to a transition state. At this point, further transition state refinements were attempted using PBE-D3/def2-SVP, but GFN2-xTB was found to significantly overestimate the length of the aryl–halide bond in the transition state, hindering convergence and making the transition state poorly transferable to higher-level theory. Hence, a more sophisticated procedure was followed for transition state refinements. Specifically, a first transition state guess was obtained by geometry optimization of the xTB-obtained transition state, constraining the carbon–halogen bond. Unconstrained geometry optimization of the resulting structures resulted in the respective oxidative addition products in all cases. Subsequently, a relaxed scan of the carbon–halogen bond to 0.8 times the initial bond length starting from the geometry obtained after constrained optimization was carried out. In addition, a partially relaxed scan of the palladium–halogen bond to 1.5 times the initial bond length was performed by constraining the carbon–halogen bond length. Relaxed geometry optimization of either the last point of the first scan or the minimum energy structure of the second scan, whichever one was lower in energy, yielded the reactant structure. Next, nudged elastic band (NEB) calculations together with a subsequent transition state optimization (keywords NEB-TS or ZOOM-NEB-TS in Orca) was performed using the reactant as starting structure and the transition state guess from the constrained geometry optimization as product structure. Final optimizations of reactants, products and transition states were performed using exact initial Hessians to remove any residual imaginary frequencies. The transition states were verified by calculating both the forward and backward intrinsic reaction coordinates and comparing the resulting endpoint structures with the respective reactant and product structures.

## Data availability

All the code used in this work can be downloaded from the following GitHub repository: https://github.com/aspuru-guzik-group/iacta (DOI: https://doi.org/10.5281/zenodo.7079486).

## Author contributions

C. L., G. P. G. and R. P. conceived the project based on an idea by C. L. C. L. designed and wrote the custom codes used in reaction discovery and data analysis. G. P. G. and R. P. provided scientific input to improve the custom codes. R. P. and C. L performed the DFT computations on oxidative addition complexes. All authors contributed to the analysis of generated data and to the writing of the manuscript.

## Conflicts of interest

There are no conflicts to declare.

## Supplementary Material

SC-013-D2SC05135D-s001
