# Supplementary material for: Guided discovery of chemical reaction pathways with imposed activation
Source: Chem Sci. 2022 Nov 10;13(46):13857–71. doi: 10.1039/d2sc05135d (PMC9710306; doi:10.1039/d2sc05135d)
Supplement: SC-013-D2SC05135D-s001 [file SC-013-D2SC05135D-s001.pdf]

Electronic Supplementary Material (ESI) for Chemical Science.
This journal is © The Royal Society of Chemistry 2022

Supplemental information for "Automatic Discovery of Chemical Reactions Using Imposed Activation"

Cyrille Lavigne,[1] Gabe Gomes,*§[1,2,a,b] Robert Pollice,*§[1,2,c] Alán Aspuru-Guzik*[1,2,3,4,5,6]

[1] Department of Computer Science, University of Toronto, 214 College St., Toronto, Ontario, M5T 3A1, Canada.
[2] Chemical Physics Theory Group, Department of Chemistry, University of Toronto, 80 St George St, Toronto, Ontario, M5S 3H6, Canada.
[3] Department of Chemical Engineering & Applied Chemistry, University of Toronto, 200 College St., Ontario, M5S 3E5, Canada.
[4] Department of Materials Science & Engineering, University of Toronto, 184 College St., Ontario, M5S 3E4, Canada.
[5] Vector Institute for Artificial Intelligence, 661 University Ave Suite 710, Toronto, Ontario, M5G 1M1, Canada.
[6] Lebovic Fellow, Canadian Institute for Advanced Research (CIFAR), 661 University Ave, Toronto, Ontario, M5G, Canada.
[a] current affiliation: Department of Chemistry, Carnegie Mellon University, Pittsburgh, Pennsylvania, 15213, United States of America.
[b] current affiliation: Department of Chemical Engineering, Carnegie Mellon University, Pittsburgh, Pennsylvania, 15213, United States of America.
[c] current affiliation: Stratingh Institute for Chemistry, University of Groningen, Nijenborgh 4, Groningen, 9747 AG, The Netherlands.
§ These authors contributed equally to this work.
Corresponding authors: gabegomes@cmu.edu, r.pollice@rug.nl, aspuru@utoronto.ca

Additional set of diverse chemical reactions

Initial tests of imposed activation (IACTA) were performed using a small but chemically diverse set of simple reactions, the results of which we describe here. All the chemical reactions shown in Figure S1 are adequately captured with minimal reaction-specific parameter choices (Table S2). The relatively small size of the molecules involved allows this entire set of reactions to be run on a single compute node in less than 5 hours.

The S_N2 reaction of methanethiol and iodomethane (Figure S1a) is readily obtained by stretching the carbon-iodine bond. Some high-energy side products include thioformaldehyde formation (by transfer hydrogenolysis) and iodomethanethiol (by a stepwise σ-bond metathesis). The aromatic Claisen[1] rearrangement (Figure S1b) is found as the sole product of stretching the allyl ether bond. Energies of the two expected transition structures, six-membered rings with chair and boat conformations, respectively, are well reproduced.[2]

In Figure S1c, the Johnson-Corey-Chaikovsky reaction[3] is obtained from stretching the methylene-sulfur bond on the ylide. The expected epoxide has by far the lowest estimated activation energy. Interesting side products include addition to the aromatic ring as well as homologations by methylene insertion into a C–H bond and by insertion into the aryl-carbon bond. This last reaction has been reported previously for benzophenones[4], with a hypothesized mechanism matching the one found here. The stretching of the sulfur-methylide (C_1 in Figure S2a) bond yields the reactive intermediate shown in Figure S2b. Insertion of C_1 between carbons marked C_2 and C_3 occurs through a four-membered cyclic transition state, displacing the sulfoxide and forming the homologation product (Figure S2c).

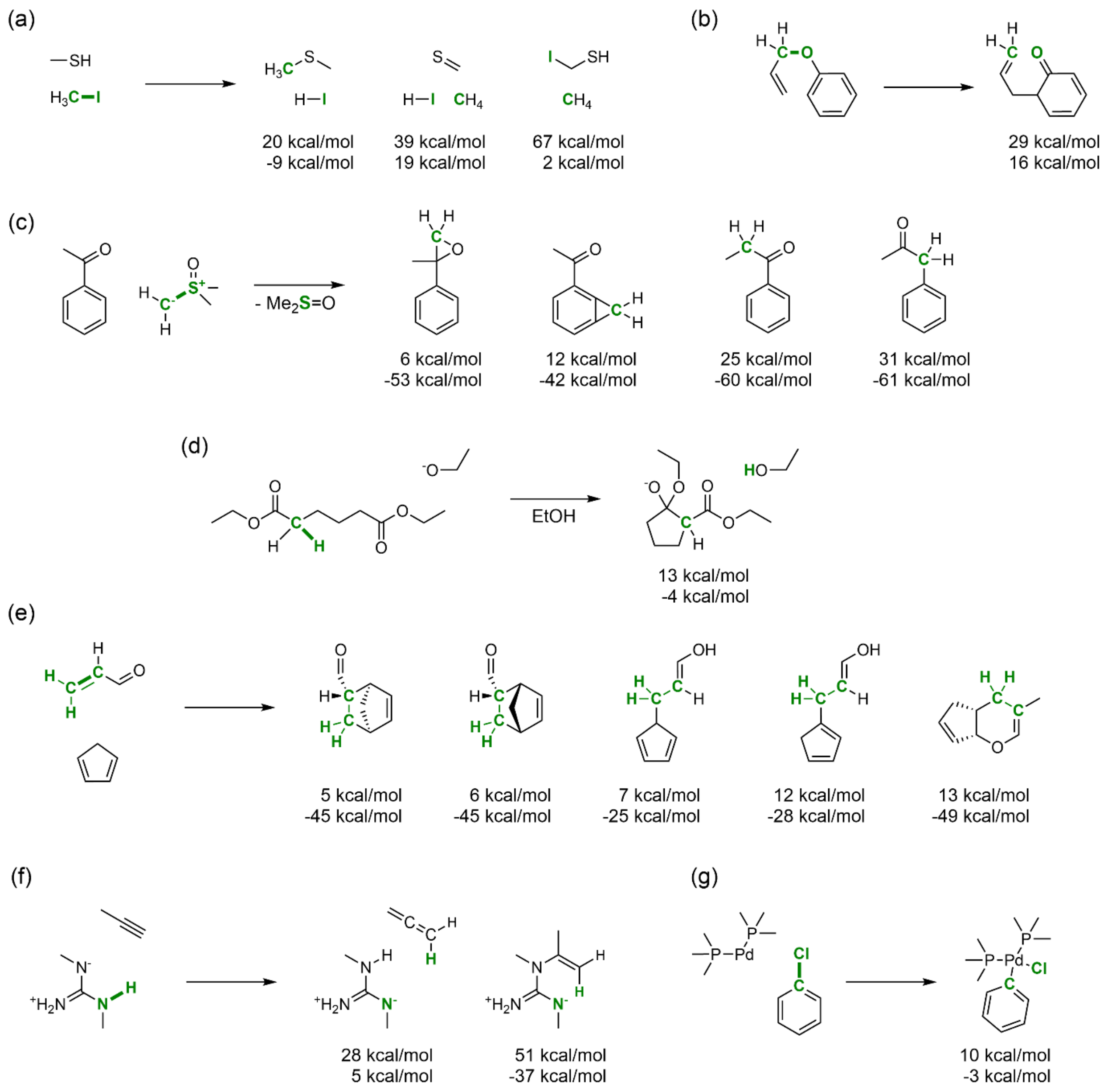

Figure S1: Selected set of diverse chemical reactions studied by imposed activation. Representative products are shown in each case, annotated with estimated activation energy (top) and reaction energy (bottom) calculated at the GFN2-xTB level of theory.[5] All results are obtained directly from the automated search without additional refinement. Activated bonds and constituent atoms are shown in green. In (e), an improper dihedral angle is activated instead of a bond length. Details of each reaction are in the text.

The Dieckmann condensation[6] in Figure S1d is found readily upon dissociation of a carbon-hydrogen bond in the presence of an ethoxide molecule. The reactants (an ester, an ethoxide molecule and two molecules of ethanol) are initially arranged without regard to the expected transition structure. The assembly is optimized to obtain the initial conformer in Figure S2d. The explicit solvent is required here to stabilize the ethoxide. The reaction search finds the Dieckmann condensation product in Figure S2e, with the

standard[7] two-step mechanism of formation of an enolate ion followed by a nucleophilic attack. Approximate transition structures are shown in Figure S2f-g. It should be noted that the initial input structure is highly dissimilar to both the structures of the transition structures and the products, illustrating that our method allows handling explicit solvent molecules without prior spatial placement by the user.

For the Diels-Alder reaction, activation can be imposed in two ways: by stretching the dienophile double bond to the length of a single bond or by pyramidalizing one of the dienophile carbons through an improper dihedral constraint applied to the two ene carbons and terminal hydrogen atoms. Both approaches yield the same products, with the pyramidalization resulting in significantly better transition structures and energies due to the asymmetric dienophile and resulting asynchronous transition state[8]. The results for this reaction are shown in Figure S1e with the atoms of the dihedral constraint marked in green. Notably, both the endo- and the exo-products are obtained, though the activation energy is approximated too poorly to reproduce experimental selectivities. Observed side reactions include Michael additions as well as an inverse electron-demand Diels-Alder reaction, with the acrolein acting as the diene and the cyclopentadiene acting as the dienophile.

The final two examples are taken from catalysis and organometallic chemistry, respectively: a base-catalysed alkyne-allene isomerization and an oxidative addition of palladium bisphosphine into a carbon-chlorine bond (Figure S1f and g). In the first case, the N-H bond on the catalyst is stretched to obtain the isomerization, and the reaction pathway and transition structure are near identical to those obtained by traditional means.[9] The oxidative addition of Pd in Figure S1g is readily found by increasing the Ph-Cl bond distance, showing that IACTA can be applied to organometallic reactions using the GFN2-xTB as level of theory.[10]

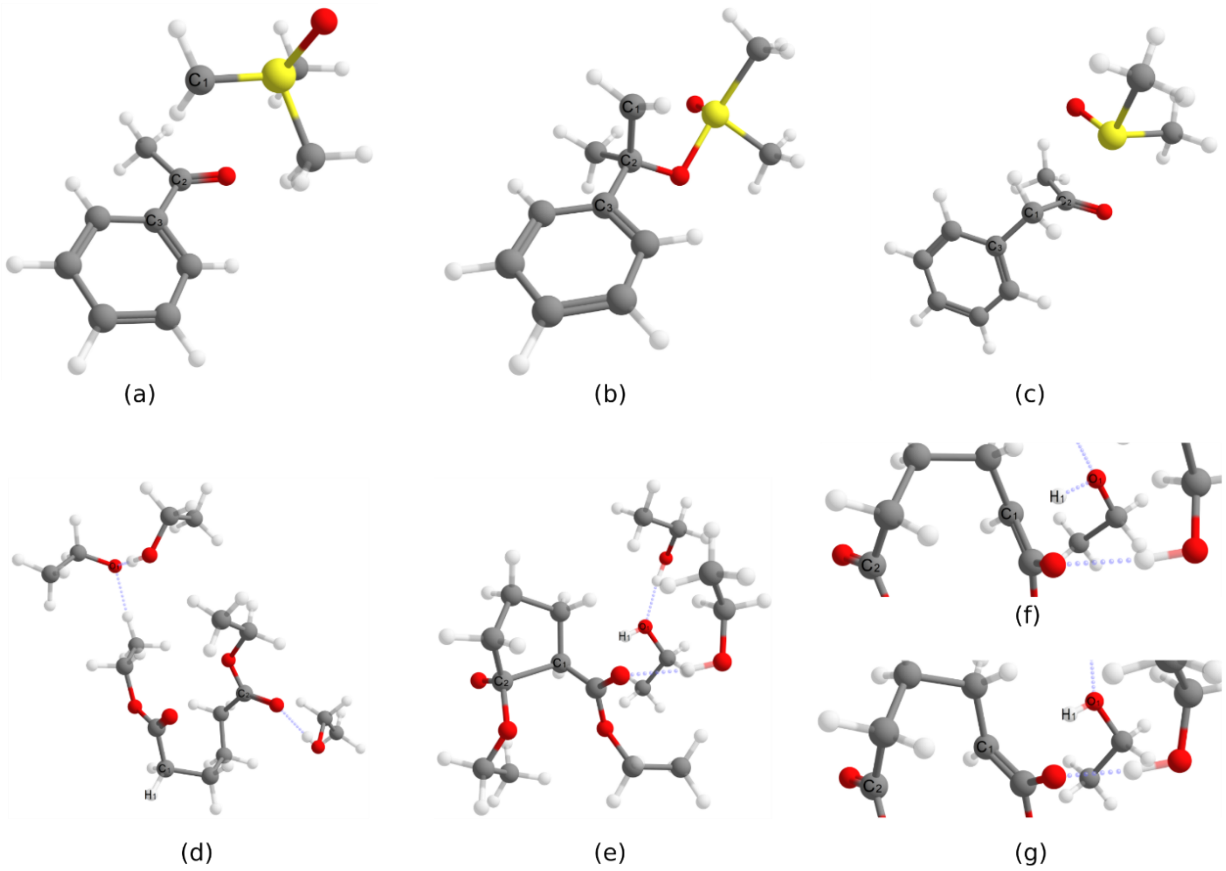

Figure S2: Detailed structures for Johnson-Corey-Chaikovsky methylene insertion and Dieckmann condensation reactions. (a-c) Reactants (a), reactive intermediate (b), and product structures (c) obtained for the homologation reaction by methylene insertion in Figure S1c. (d-g) Input structure (d) and products (e) for the Dieckmann condensation in Figure S1d and transition structures of the two steps, formation of an enolate (f) and nucleophilic attack (g), as obtained from a single relaxed scan.

Algorithmic details and numerical parameters.

Table S1 lists default numerical parameters used for the computations described in this article. Unless otherwise noted, these values are used throughout. Reaction-specific parameters (specifically, those that differ from defaults) are shown in Table S2. Here, we described in more details the implemented algorithm.

All runs are initialized by performing a metadynamics propagation (MTD1 in Table S1) filtering all the resulting structures and selecting the bottom 10% based on energy. This is done with the activating coordinate $q^{\ddagger}$ constrained to its initial value.

Relaxed scans are performed from the initial value of ${q_i}^{\ddagger}$ to its final value ${q_f}^{\ddagger}$ over a uniformly discretized grid of values. Metadynamics searches for activated conformers (MTD2) are performed for points $j = M_L$ to M_U of the scans with $q^{\ddagger}$ constrained to its value at that point, denoted ${q_j}^{\ddagger}$. Each metadynamics search is initialized from one of the structures obtained in the initialization.

Structures obtained from metadynamics propagation at point j are optimized with $q^{\ddagger}$ constrained to $q_j{}^{\ddagger}$ and screened for duplicates. Those structures with energy below two thresholds, a local threshold $E < \min_j E + \Delta_l$ (where the minimum is over structures from the same metadynamics propagation) and a global threshold $E < E_0 + \Delta_g$ (where E_0 is the energy of the reactant conformer) are selected as starting points for reaction scans. The reaction scans are relaxed scans from $q_j{}^{\ddagger}$ to $q_f{}^{\ddagger}$ and to $q_i{}^{\ddagger}$, yielding new product and reactant structures.

Finally, all reaction scans are searched for potential stable structures, which are local energy minima on the $q^{\ddagger}$ axis. Those stable points are optimized without any constraining potentials. The overall scan is then analyzed for reactions, as described in the methods section.

Table S1 Default parameters used in computations.

xtb parameters

Parametrization	GFN2
Electronic temperature	300K
Cavity potential †	Logfermi, spherical with diameter $d_{\mathrm{reactants}} + 4\text{Å}$

Metadynamics propagation (MTD1)

Propagation time	0.5 ps per atom
Bias potential ‡	$k = 0.20 E_H,\ \alpha = 0.2/\text{Å}^2$<br><br>Structure saved every 100 fs<br>Biasing by previous 10 saved structures
Timestep §	5 fs
SHAKE constraints §	All atoms
Structures kept	Bottom 10% in energy after filtering

Relaxed scans

Number of scan points between q_i‡ and q_f‡	$N_S = 50$
Constraint force constant **	$k = \text{computed or 1 kcal/mol}$
Optimization tolerance	"normal"<br>$\Delta E = 5 \times 10^{-6} E_H, \text{max grad} = 10^{-3} E_H/\alpha$

Metadynamics propagation (MTD2)

Propagation time	0.1 ps per atom
Bias potential††	$k = 0.20\ E_H, \alpha = 0.8/\text{Å}^2$<br>$k = 0.20\ E_H, \alpha = 0.2/\text{Å}^2$<br>$k = 0.05\ E_H, \alpha = 0.8/\text{Å}^2$<br>$k = 0.05\ E_H, \alpha = 0.2/\text{Å}^2$<br><br>Structure saved every 100 fs<br>Biasing by previous 10 saved structures
Timestep	2 fs
SHAKE constraints	None
Performed at...	... every point between $M_L = 0$ and $M_U = 0.5 \times N_s$

Conformer filtering ‡‡

Optimization tolerance	"tight"<br>$\Delta E = 10^{-6} E_H, \text{max grad} = 8 \times 10^{-4} E_H/\alpha$
Similarity thresholds	RMSD = 0.4 Å<br>Energy = 1.0 kcal/mol
Local threshold Δ_l	12 kcal / mol
Global threshold Δ_g	60 kcal/mol above reactants

† For metadynamics propagations, a spherical constraining potential[11] is added to stop molecules in the reacting system from separating. $d_{\mathrm{reactants}}$ is the maximum atom pair distance of the reactant structure.

‡ The metadynamics potential has the following form $V_{\mathrm{bias}} = \sum_i k \exp\left(-\alpha \Delta_i^2\right)$ where the sum is over contributing structures and Δ_i is the RMSD between contributing structure i and the current structure.

§ If the initial metadynamics propagation fails to converge, it is restarted with a timestep of 2 fs and no SHAKE constraints.

** For bond stretches, the force constant is computed from an estimated bond force constant, computed from a quadratic fit of the energy from a five-point relaxed scan around the equilibrium bond length.

†† Four metadynamics propagations are done,[11] one for each pair of values k, α.

‡‡ Structures from all metadynamics calculations are first optimized and then duplicated structures are removed based on similarity, followed by elimination using energy thresholds.

Table S2 Parameters used for specific reactions described in this article.

		Activation coordinate scans	Parameters changed from Table S1
Reactions of 2-iodobutane		$q_0{}^{\ddagger} \rightarrow 6.0$ Å	GBSA solvent model for THF<br>$N_S = 100$
Cyclization cascade		1.5 Å $\rightarrow$3.0 Å	$N_S = 100$<br>$M_U = 0.3 \times N_S$
Acrolein water-mediated 1,4-addition		$q_0{}^{\ddagger} \rightarrow 1.6$ Å	$N_S = 100$<br>$M_U = 0.3 \times N_S$<br>MTD2 time per atom = 0.5 ps
Oxidative additions of drug candidates		$q_0{}^{\ddagger} \rightarrow 3.5$ Å	$N_S = 200$<br>$M_L = 0.06 \times N_S,\ M_U = 0.12 \times N_S$
Reactions in Figure S1			
	(a)	$q_0{}^{\ddagger} \rightarrow 6.0$ Å	GBSA solvent model for methanol
	(b)	$q_0{}^{\ddagger} \rightarrow 4.0$ Å	
	(c)	$q_0{}^{\ddagger} \rightarrow 4.0$ Å	
	(d)	$q_0{}^{\ddagger} \rightarrow 3.5$ Å	
	(e)	$q_0{}^{\ddagger} \rightarrow 120°$	$M_L = 0.30 \times N_S,\ M_U = 0.80 \times N_S$
	(f)	$q_0{}^{\ddagger} \rightarrow 3.0$ Å	
	(g)	$q_0{}^{\ddagger} \rightarrow 3.5$ Å	

$q_0{}^{\ddagger}$ denotes the equilibrium value of the activation coordinate.

References

(1) Claisen, L. Über Umlagerung von Phenol-Allyläthern in C-Allyl-Phenole. *Berichte Dtsch. Chem. Ges.* **1912**, *45* (3), 3157–3166. https://doi.org/10.1002/cber.19120450348.

(2) Yamabe, S.; Okumoto, S.; Hayashi, T. Transition Structures for the Aromatic Claisen Rearrangements by the Molecular Orbital Method. *J. Org. Chem.* **1996**, *61* (18), 6218–6226. https://doi.org/10.1021/jo9522419.

(3) Corey, E. J.; Chaykovsky, M. Dimethyloxosulfonium Methylide ((CH3)2SOCH2) and Dimethylsulfonium Methylide ((CH3)2SCH2). Formation and Application to Organic Synthesis. *J. Am. Chem. Soc.* **1965**, *87* (6), 1353–1364. https://doi.org/10.1021/ja01084a034.

(4) Chittimalla, S. K.; Chang, T.-C.; Liu, T.-C.; Hsieh, H.-P.; Liao, C.-C. Reactions of 2-Hydroxybenzophenones with Corey–Chaykovsky Reagent. *Tetrahedron* **2008**, *64* (11), 2586–2595. https://doi.org/10.1016/j.tet.2008.01.024.

(5) Bannwarth, C.; Ehlert, S.; Grimme, S. GFN2-XTB—An Accurate and Broadly Parametrized Self-Consistent Tight-Binding Quantum Chemical Method with Multipole Electrostatics and Density-Dependent Dispersion Contributions. *J. Chem. Theory Comput.* **2019**, *15* (3), 1652–1671. https://doi.org/10.1021/acs.jctc.8b01176.

(6) Dieckmann, W. Zur Kenntniss Der Ringbildung Aus Kohlenstoffketten. *Berichte Dtsch. Chem. Ges.* **1894**, *27* (1), 102–103. https://doi.org/10.1002/cber.18940270126.

(7) Schaefer, J. P.; Bloomfield, J. J. The Dieckmann Condensation (Including the Thorpe-Ziegler Condensation). In *Organic Reactions*; John Wiley & Sons, 2011; pp 1–203. https://doi.org/10.1002/0471264180.or015.01.

(8) Loncharich, R. J.; Brown, F. K.; Houk, K. N. Transition Structures of the Diels-Alder Reaction of Butadiene with Acrolein. *J. Org. Chem.* **1989**, *54* (5), 1129–1134. https://doi.org/10.1021/jo00266a026.

(9) dos Passos Gomes, G.; Morrison, A. E.; Dudley, G. B.; Alabugin, I. V. Optimizing Amine-Mediated Alkyne–Allene Isomerization to Improve Benzannulation Cascades: Synergy between Theory and Experiments. *Eur. J. Org. Chem.* **2019**, *2019* (2–3), 512–518. https://doi.org/10.1002/ejoc.201801052.

(10) Bursch, M.; Neugebauer, H.; Grimme, S. Structure Optimisation of Large Transition-Metal Complexes with Extended Tight-Binding Methods. *Angew. Chem.* **2019**, *131* (32), 11195–11204. https://doi.org/10.1002/ange.201904021.

(11) Grimme, S. Exploration of Chemical Compound, Conformer, and Reaction Space with Meta-Dynamics Simulations Based on Tight-Binding Quantum Chemical Calculations. *J. Chem. Theory Comput.* **2019**, *15* (5), 2847–2862. https://doi.org/10.1021/acs.jctc.9b00143.
